# A New Source of Elemol Rich Essential Oil and Existence of Multicellular Oil Glands in Leaves of the *Dioscorea* Species

**DOI:** 10.1155/2013/943598

**Published:** 2013-12-23

**Authors:** Joy I. Odimegwu, Olukemi Odukoya, Ritesh K. Yadav, C. S. Chanotiya, Steve Ogbonnia, Neelam S. Sangwan

**Affiliations:** ^1^Metabolic and Structural Biology Department, CSIR-Central Institute of Medicinal and Aromatic Plants (CIMAP), Lucknow, UP 226015, India; ^2^Department of Pharmacognosy, Faculty of Pharmacy, College of Medicine Campus, University of Lagos, Lagos, Nigeria

## Abstract

*Dioscorea* species is a very important food and drug plant. The tubers of the plant are extensively used in food and drug purposes owing to the presence of steroidal constituent's diosgenin in the tubers. In the present study, we report for the first time that the leaves of *Dioscorea composita* and *Dioscorea floribunda* grown under the field conditions exhibited the presence of multicellular oil glands on the epidermal layers of the plants using stereomicroscopy (SM) and scanning electron microscopy (SEM). Essential oil was also isolated from the otherwise not useful herbage of the plant, and gas chromatographic-mass spectroscopy analysis revealed confirmation of the essential oil constituents. Out of the 76 compounds detected in *D. floribunda* and 37 from *D. composita* essential oil, major terpenoids which are detected and reported for *Dioscorea* leaf essential oil are **α**-terpinene, nerolidol, citronellyl acetate, farnesol, elemol, **α**-farnesene, valerenyl acetate, and so forth. Elemol was detected as the major constituent of both the *Dioscorea* species occupying 41% and 22% of *D. Floribunda* and *D. composita* essential oils, respectively. In this paper, we report for the first time *Dioscorea* as a possible novel bioresource for the essential oil besides its well-known importance for yielding diosgenin.

## 1. Introduction

*Dioscorea* commonly referred to as yam is a monocotyledonous tuber plant of the family Dioscoreaceae with about 600 species recorded so far. Different species originate from different parts of the world: Africa, Asia, the Caribbean's South America, and the South Pacific islands, and so forth. The dominant zone for yam production in the world is in West Africa [1], where about 48 million tonnes (about 93% of the world's production) are cultivated on 4 million hectares annually, and mainly in five countries—Benin, Côte d'Ivoire, Ghana, Nigeria, and Togo (FAO, 2005) feeding about 100 million people in the tropics [1]. The more domesticated and cultivated ones are native to Africa [2]. Yams, especially the wild species: *D. floribunda*, *D. composita*, *D. dumetorum*, and *D. villosa*, and so forth are essentially orphan crops and are therefore classified as neglected and under-utilised species (NUS) due to the fact that though they are rich in nutrients, phytochemicals [3], they are also important in strategies to alleviate biotic and abiotic stresses linked to climate change and are important in traditional pharmacology but yet are adapted to low input agriculture. Yam extracts are emerging as good candidates for the treatment of certain illnesses, for example, menopausal symptoms and some forms of cancer [4]. It is also suggested that yam might reduce the risk of breast cancer and cardiovascular diseases in postmenopausal women [4]. Essential oils usually occur as mono- and sesquiterpenoids in plants and are important commodities and used for centuries medicinally and as cosmetics [5]. The oil glandular trichomes are the primary sites of essential oil biosynthesis [6–10]. Multicellular peltate and single celled capitate glands contribute significantly to the biosynthesis and accumulation of essential oil content [8, 9, 11]. Essential oils with therapeutic effects have been reported in tubers of some yam species: *D. alata*, rhizomes of *D. japonica* [12]. Metabolic engineering of monoterpene biosynthesis in the model plant tomato and peppermint has resulted in increase in yield and compositional improvement of the essential oil and also provided strategies for manipulating flavor and fragrance production, as well as plant defense [5, 8, 13, 14]. The qualitative, quantitative, and interspecific variations in essential oil constituents have been reported to occur for several plant species in earlier reports [15–17].

**Figure 1:**
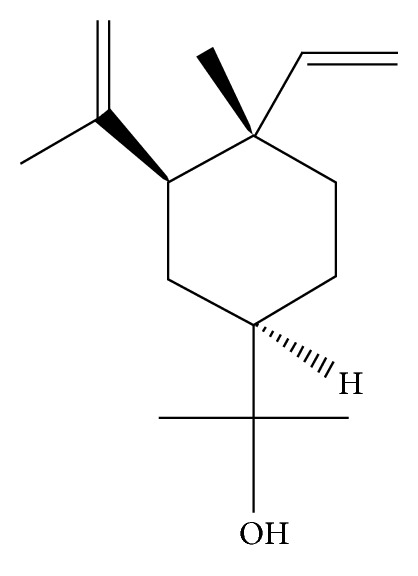
Structure of elemol.

In the present study, we attempted to determine if the diosgenin yielding *Dioscorea* species possess essential oil in their aerial parts and also the nature and contents of essential-oil and oil glands bearing the essential oil in two species of *Dioscorea*, namely, *D. composita* and *D. floribunda* leaves and vines. The study will aid in the search for and development of novel elemol (Figure 1) rich essential oil of *Dioscorea* chemotypes and/or species.

## 2. Materials and Methods

### 2.1. Plant Materials

The plants of *Dioscorea composita* and *D. floribunda* were obtained from the experimental farm at Central Institute of Medicinal and Aromatic Plants (CSIR-CIMAP, Lucknow) in May 2010, Lucknow (26.5°N latitude, 80.5°E longitude), 120 m above mean sea level, subtropical, semiarid zone for the studies.

### 2.2. Stereomicroscopic and Scanning Electron Microscopic Analysis of Leaf

For stereomicroscopic observation and analysis of glandular trichomes from two species of *Dioscorea,* namely, *D. composita* and *D. floribunda*, fresh leaf samples were taken. Both the adaxial and abaxial leaf surfaces were processed as described earlier [8]. The processed leaf sample was mounted on clean labelled microscope slide. Leaf impression is studied under a Leica model SM starting at magnification from 100x to 400x for quantitative and qualitative analysis. For SEM analysis, the leaf and vine samples were immersed in 2.5% glutaraldehyde in 0.1 M phosphate buffer pH 7.4 (with 0.02% Triton X-100) overnight at 4°C. Three washes (each after 5 minutes duration) in 0.1 M phosphate buffer pH 7.4 were given to clean the material. Subsequently, dehydration was followed as 1X 10 minutes in 10%, 30%, 50%, 70%, 95%, and 100% ethanol. Finally samples are left in the 100% ethanol solution. Samples were air-dried under a light bulb and were mounted onto metal stubs with double-sided carbon tape. Samples were then subjected to automated sputter coater which applies a thin layer of metals, gold and palladium over them for about 10 minutes.

### 2.3. Extraction and Gas Chromatographic Analysis of Essential Oil

30 g fresh leaves were collected over ice and hydro-distilled using Clevenger type apparatus to obtain the essential oil for GC/MS analysis as described earlier [8]. The conditions for GC-MS were as follows: a set of sample (*D. floribunda and D. composita*) specific conditions for the GC-MS, utilized a Perkin Elmer AutoSystem XL GC interfaced with a TurboMass Quadrupole mass spectrometer fitted with an Equity-5 fused silica capillary column (60 m × 0.32 mm i.d., film thickness 0.25 *μ*m; Supelco Bellefonte, PA, USA). The oven temperature program ranged from 70°C to 250°C, programmed at 3°C/min, with initial and final hold time of 2 min, carrier gas: He at 10 psi constant pressure, a split ratio of 1 : 40; injector, transfer line, and source temperatures were kept at 250°C; ionization energy 70 eV; mass scan range 40–450 amu. Characterization was achieved on the basis of retention time, elution order, coinjection with standard elemol in GC-FID capillary column (Aldrich and Fluka), mass spectra library search (NIST/EPA/NIH version 2.1, and Wiley registry of mass spectral data 7th edition).

## 3. Results and Discussion

### 3.1. Stereomicroscopic (SM) and Scanning Electron Microscopic (SEM) Analysis of *Dioscorea* Species

**Figure 2:**
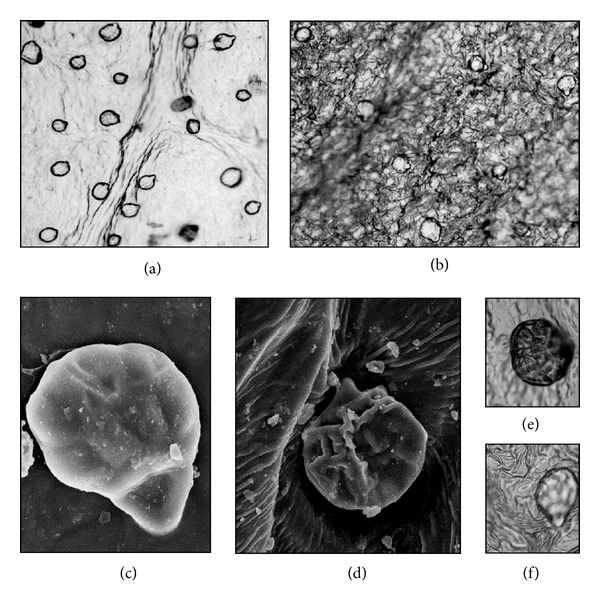
(a) and (b): stereomicroscopic picture of *D. composita *and* D. floribunda *essential-oil glandular trichomes at 100x, respectively. (c) and (d): scanning electron microscopic picture of *D. composita *and* D. floribunda *essential-oil glandular trichomes at 2.74 kkX, respectively. (e) and (f): 400x stereomicroscopic views of magnifications of essential-oil glandular trichomes of *D. composita* and *D. floribunda,* respectively. All were viewed abaxially.

The multicellular peltate oil glands were observed on both *D. composita* and *D. floribunda*, with the gland head supported by a stalk cell protruding from the basal cells of the leaves and vines (Figure 2). The population of oil glands on the abaxial surface of *D. composita* appears more than that on *D. floribunda* on the basis of the observations on the oil gland count under microscope. The leaves from* D. composita *yielded about 0.014% (w/w), while *D. floribunda *yielded slightly higher quantity (0.016% w/w) of essential oil.

### 3.2. Essential Oil Composition of *Dioscorea* Species

A total of 76 compounds were detected from the GC-MS of *D. floribunda* and most were in trace amounts, while in *D. composita*, a total of 37 compounds were detected, and like in *D. floribunda* most of the constituents were present in trace amounts. Mostly sesquiterpenes were detected (85% of essential oil) in *D. composita* and (48% in essential oil) in *D. floribunda* essential oils. The overall monoterpene content was detected in traces in both of the *Dioscorea* species. The % of elemol in *D. composita* was recorded as 41.02% of total terpenoid, while it is 22.88% of total terpenoid contents in case of *D. floribunda *leaves (Tables 1 and 2). However, elemol remained as a major oil constituent in both of the *Dioscorea* species. This communication describes evidence of multicellular peltate oil glands on the leaves and vines of the *Dioscorea* species and also exhibits existence of novel oil constituent in its essential oil. The anatomical studies with SM and SEM clearly show the presence of essential oil glands in both the species. Also, GC-MS results revealed elemol to be the most abundant essential oil constituent. This compound is a sesquiterpene alcohol with a green woody sweet odour. Of the terpenoids, only members of the mono-(C10) and sesquiterpenoid (C15) classes are sufficiently volatile [18]. Elemol has been reported as an important terpenoid constituent with insecticidal properties [19]. The SM and SEM reveal higher abundance of the oil glands on the abaxial leaf surface of both species and more so in *D. composita* than in *D. floribunda *leaves. It is speculated that, evolutionarily, the peltate glands might represent an advanced structure of ecological significance in accumulation and functionality of volatile secondary metabolites [6]. Lack of programmed gland/cuticular dehiscence in capitate glands together with their limited oil-sequestration capacity might put them as a sort of “primitive” oil glands or secretory trichomes which originated from nonglandular trichomes in a phylogenetic sequence [6]. As an evolutionary link in the transition from capitate to peltate glands, some plant species possess capitate glands with more than one secretory head cell, and capitate glands in some plant species possess exocytotic mechanisms of emission of volatiles through rudimentary rupture mode [19]. It has been reported that the **β**-elemol and **α**-terpineol achieved 100% termite mortality at the dosage of 1 mg/g after 7 d of testing [20, 21]. Thus, **β**-elemol and **α**-terpineol have been shown to possess commendable antitermitic activity [20, 21].

The novel results from this study will facilitate the development of *Dioscorea* as a high value plant and encourage further research on it for increment of the essential oil through genetic engineering or otherwise in order to help the people living in areas where it grows abundantly and who also happen to be one of the world's poorest peoples [14]. The study will aid in the search and development of novel elemol rich essential oil of *Dioscorea* chemotypes and/or species. It may also be possible to utilize the waste herbage for the antitermite molecule elemol which is present in high proportions after the *Dioscorea* tubers are taken out.

## 4. Conclusions

This communication describes for the first time evidence of multicellular peltate oil glands on the leaves of the *Dioscorea composita* and *D. floribunda *and also exhibits existence of novel oil constituent elemol in its essential oil by GC-MS. Elemol has been reported as an important terpenoid constituent with insecticidal and antitermite properties. *D. composita* with about 41% of elemol in its essential oil could be one of the best sources for elemol rich essential oil, if the efforts are made to express essential oil in leaves in higher amounts. The results reveal that *Dioscorea* in addition to its well-known usage of its tubers as food and drug crop can also be an important elemol rich essential oil bearing plant.

## Figures and Tables

**Table 1:** Major essential oil constituents, their retention time, and MS data for *Dioscoria composita *and *Dioscorea floribunda*. Different libraries such as Wiley, Nbs, and Nist were used for identification of compounds.

Plant	Peak no	Lib	Time (mins)	Compound	MS
*D. composita *	2	W	9.85	$\overset{´}{\alpha }$ terpinene	39,44,65,77,91,93,105,121,136,137
	3	W	9.85	$\overset{´}{\alpha }$ terpinene 1,3-cyclohexadiene, 1 methyl-4-methylethy	27,39,77,91,93,105,121,136,137
	3	N	30.6	Butylated hydroxytoluene	29,41,57,91,105,115,145,177,205,220,221
	4	W	30.6	Phenol 1,6 Bis (1,1-dimethylethyl)-4-methyl-cas	41,57,81,105,145,177,205,220,221
	4	W	38.87	Nerolidol	53,55,69,79,93,119,133,161,204,205
	4	W	37.57	Citronellyl acetate	43,67,69,81,95,123,138
	5	W	9.85	$\overset{´}{\alpha }$ terpinene 1,3-cyclohexadiene, 1 methyl-4-methylethyl-4	27,39,77,91,93,105,121,136,137
	5	W	38.87	Farnesol	39,41,55,69,93,107,119,133,161,204
	5	Nbs	37.35	Dodecyl acrylate	41,55,56,73,83,97,111,140,168
	5	Nbs	37.57	Doecadien-1-ol	41,55,69,81,95,123,181,224
	6	W	37.35	2- Decenal	41,43,55,57,70,83,97
	6	W	37	3-Isopropenyl	43,55,77,91,105,121,131,161,189,204,205
	6	W	37.17	Selina-3,7 (11)-Diene	29,41,55,81,91,107,122,133,161,189,204,205
	10	W	32.47	Elemol	41,67,68,81,93,107,121,161,189,204,222
	11	W	32.47	*β*-elemene	51,53,67,81,93,107,147,161,189,190
	15	Nbs	39.75	*α*-farnesene	29,41,55,69,93,107,119,135,161,204
	16	N	39.75	1,3,6,10-Dodecatetraene	41,55,69,93,119,120,161,189,204

*D. floribunda *	3	W	38.9	Farnesol	39,41,55,69,93,107,119,133,161,204
	3	W	36.62	Torreyol	39,41,43,81,93,105,119,133,161,162,204,205
	4	Nbs	38.9	2,6,10-Dodecatrien-1-ol	39,41,55,69,93,107,119,133,161,204
	4	W	36.62	Isoledene	39,41,55,81,91,105,119,133,161,162,204,205
	4	W	34.72	Aromadendrene	41,55,69,91,93,105,133,161,189,204,205
	4	W	37.17	Selina-3,7(11)-diene	29,41,55,81,91,107,122,133,161,189,204,205
	4	W	37.35	Dodecanol	41,43,55,69,70,83,97,111,140,168,169
	4	N	9.87	Cyclohexene	27,39,53,79,91,93,105,121,136,137
	5	N	34.72	2,4,4-Trimethyl-3B hydroxymethyl	67,69,77,91,107,121,135,189,207
	5	W	42.92	1,6,6-Trimethyl-10 oxatricyclo [8.1.0]undec-3yn-2-ol	40,41,43,77,91,95,121,123,177,220
	5	W	9.87	*α*-terpinene	41,77,91,93,119,121,136,137
	7	W	36.2	Cadinene	27,41,55,81,91,105,119,133,161,162,189,204,205
	7	W	46.12	1,H-dimethyl-7	41,59,77,91,105,131,159,162,202,203
	8	W	36.47	Torreyol	39,41,43,81,93,105,119,133,161,162,204,205
	8	W	46.12	1,8-Anhydro-cis-*α*-copaene-8-ol	41,77,91,119,145,159,187,202,203
	9	W	36.47	4-Iodobis bicycle[2.2.1] hexane	77,79,91,105,133,161,288,289
	10	W	36.97	*α*-cadinol	71,81,95,105,121,134,161,162,204,205,223
	10	W	32.52	Elemol	41,67,68,81,93,107,121,161,189,204,222
	10	W	45.52	Deoxycapsidiol	41,55,77,91,105,119,159,173,187,202,220
	11	W	42.8	7-1-Methyl-ethenyl-1-hydroxy-1,4-dimethyl	41,43,79,91,105,145,162,173,202,205,223
	11	N	32.52	*γ*-Elemene	39,41,55,67,79,93,121,136,161,189,204
	11	N	45.52	1H-cyclopropa [A] naphthalene	41,43,55,77,91,131,159,187,202
	13	W	44.82	Valerenyl acetate	41,43,55,79,91,105,107,145,160,161,187,202,203

**Table 2:** Essential oil constituents and their percentages in oil from *Dioscorea composita* and *Dioscorea floribunda*.

RT	Constituent name	Molecular formula	Type of constituent	% in *D. composita *	% in *D. floribunda *
9.87	Alpha terpinene	C_10_H_16_	Monoterpene	0.45	0.06
30.6	Butylated hydroxytoluene	C_15_H_24_O	Sesquiterpene	7.57	2.68
32.47	Elemol	C_15_H_26_O	Sesquiterpene	41.02	22.88
32.52	Elemene	C_15_H_24_	Sesquiterpene	0.33	0.31
34.72	Aromadendrine	C_15_H_24_	Sesquiterpene	0.82	0.04
36.2	Naphthalene	C_15_H_26_	Sesquiterpene	3.71	2.48
36.45	Torreyol	C_15_H_26_O	Sesquiterpene	3.91	2.45
36.62	Alpha copaene	C_15_H_24_	Sesquiterpene	0.44	0.56
36.97	Azulene	C_15_H_24_	Sesquiterpene	2.12	2.31
37.17	Selinene	C_15_H_24_	Sesquiterpene	4.32	9.57
37.35	Dodecyl acrylate	C_15_H_28_O_2_	Sesquiterpene	0.95	0.19
37.57	Citronellyl acetate	C_12_H_22_O_2_	Monoterpene	0.32	0.28
38.87	Farnesol	C_15_H_26_O	Sesquiterpene	14.81	0.65
39.75	Farnesene	C_15_H_24_	Sesquiterpene	0.64	0.18
42.80	Gamma-costol	C_15_H_24_O	Sesquiterpene	1.51	1.45
42.92	Beta-costol	C_15_H_24_O	Sesquiterpene	0.76	0.78
44.82	Valerenyl acetate	C_17_H_26_O_2_	Sesquiterpene	0.69	0.55
45.52	1-Deoxycapsidiol	C_15_H_24_O	Sesquiterpene	0.58	0.43
46.12	1,8-Anhydro-cis-alpha-copaene-8-ol	C_15_H_22_	Sesquiterpene	0.49	0.66
